# A treatise for a new philosophy of chiropractic medicine

**DOI:** 10.1186/s12998-017-0138-y

**Published:** 2017-03-06

**Authors:** Timothy A. Mirtz

**Affiliations:** Department of Secondary and Physical Education, Bethune-Cookman University, 640 Dr. Mary McLeod Bethune Blvd., Daytona Beach, FL 32114 USA

**Keywords:** Philosophy, Chiropractic medicine, Principles, Russell Kirk, Economics, Collectivism, Politics, Government, Regulation

## Abstract

**Background:**

The philosophy of chiropractic has been a much debated entity throughout the existence of the chiropractic profession. Much criticism has been passed upon the historical philosophy of chiropractic and propagated by contemporary adherents. To date, a new philosophy has not been detailed nor presented that demonstrates principles by which to follow.

**Aim:**

The purpose of this paper is to expand upon the work of Russell Kirk (b.1918, d. 1994), an American political theorist, as a basis for principles to guide the formation of a philosophy of chiropractic medicine (PCM). Each of Kirk’s principles will be explained and expounded upon as applicable to a PCM. The addition of the term “medicine” to chiropractic is indicative of a new direction for the profession.

**Discussion:**

The ten principles that provide a foundation for a PCM include: (a) moral order, (b) custom, convention and continuity, (c) prescription, (d) prudence, (e) variety, (f) imperfectability, (g) freedom and property linkage, (h) voluntary community and involuntary collectivism, (i) prudent restraints upon power and human passions, and (j) permanence and change. Each of these principles offers not a dogmatic approach but provides insight into the application of chiropractic medicine to the entire station of the patient and society at large especially that of the economic, social and political. These principles provide direction in not only the approach to the doctor-patient encounter but can be used to visualize the wider world and its potential impact. Instead, these principles examine many tangential issues worthy of discussion that may impact health, social, political, and economic policy and how the chiropractic profession can approach these issues.

**Conclusion:**

This paper provides the initial steps in formulating a PCM using principles from a sociological, political and economic standpoint which may impact on how chiropractic medicine approaches the patient and society in totality. In addition, these principles provide the necessary first steps in the arena of the social, political and economic aspects and how chiropractic medicine can advance.

## Background

For the past 120 years a consistent, a coherent or defensible philosophy of the chiropractic profession has failed to be projected [1]. Throughout the profession’s history a multitude of efforts to establish an operational philosophy have occurred. From the traditional Palmer philosophical dictates to today’s evidence-based paradigm, much discussion and dissent has been appreciated. Despite ongoing debate, a philosophy of chiropractic medicine (PCM) has not emerged that forwards principles for establishing a pro-growth attitude. Nor has a philosophy emerged that can adequately embrace the social, political and economic aspects of society. Professional infighting, based no less on philosophy, has genuinely harmed efforts of having a greater impact on society. Yet Coulter [2] noted that a philosophy of chiropractic can exist. Despite this, much of what has been forwarded as a philosophy has been met with much criticism [3–9].

### Operational definitions for chiropractic medicine

An operational definition of what is a philosophy of chiropractic medicine can be defined. A philosophy of chiropractic medicine (PCM) is operationalized as a *philosophy of a health care profession that embraces the patient as the focal point of care and advances itself within the larger health care community and society en masse through health*, *political*, *social and economic policy*. The addition of the term “medicine” is used to fully describe chiropractic’s potential as an emerging health care enterprise and offers more clarity. The clarity can be appreciated in that it is not mired with past items which have failed to propel the profession forward i.e. subluxation, innate, etc. From this a definition of chiropractic medicine emerges. Chiropractic medicine is defined as *the knowledge*, *skills and abilities in the diagnosis*, *treatment*, *rehabilitation and prevention of carefully selected neuro*-*musculo*-*skeletal conditions with the proper use of the modalities of manipulative therapy*, *physiotherapeutic and pharmacological measures under the auspices of an evidence*-*based medicine paradigm*. These operational definitions provide for the wider discussion of why principles are needed to fully implement such. Despite the contentious topic of pharmacology rights in chiropractic practice there has been a push for expanding the scope of practice to include limited drug prescription [10–12].

### Calls for reform

With any development of a PCM the current status of the profession needs to be considered. Murphy et al. [13, 14] delineated nine areas of much needed reform in North America. These areas of reform included (a) education, (b) research, (c) regulatory, (d) practice management, (e) public image, (f) marketing, (g) professional interaction, (h) reimbursement, and (i) Medicare [13, 14]. These areas of reform prompted Murphy et al. [13, 14] to suggest that immediate action was needed. Walker [15] offered a ten-point plan to legitimize the chiropractic profession. These included (a) improvement of pre-professional education, (b) establishment of a progressive identity, (c) development of a generalized special interest, (d) marginalization of elements of nonsense, (e) a pro-public health stance, (f) support for legitimate chiropractic organizations, (g) clinical practice improvement, (h) embracing of evidence-based practice, (i) support of research and (j) individual leadership to effect change. The medical profession has noted issues that have pertinence for chiropractic medicine. Ausman [16], Editor-in-Chief of *Surgical Neurology International*—*USA* illustrated five items of political interest/concern for physicians: (a) the progressive centralization of the control of medicine by large organizations and the government, (b) the socialization of America and an entitlement mentality, (c) the loss of individual freedom, (d) the deterioration of the economy and (e) the loss of ethics and morality. These concerns expressed by Ausman [16], Murphy [13, 14] and Walker [15] need to be heeded by the chiropractic profession. Yet, there is a need for principles by which to meet these reform challenges.

The ability to address society as a whole leaves wide-open the likelihood of expanding upon great questions that may answer the calls for reform by Murphy [13, 14], Walker [15] and Ausman [16]. Such questions can be expounded upon and consist of “how can the future of the profession be a part of the social fabric of the wider culture?”, “how can the profession impact the larger society morally and ethically?”, “how can the profession contribute to the overall well-being of the country at-large socially, politically and economically?” and “how can the profession impact not only in health but in matters that affect and empower everyday individuals for work, prosperity, home and family?” If the profession wishes to advance it may need to re-think its current position and look to a differing model. In other words, instead of looking for how chiropractic medicine can benefit itself and the individual patient, the profession may need to look to how it can impact society.

### Current models

But what is a current model for a philosophy of chiropractic? Coulter [2] described a philosophy of chiropractic as having five components. These components included (a) vitalism (body’s self-repair mechanism; an unmeasurable metaphysical force in living material), (b) holism (the sum is greater than the parts), (c) naturalism (natural treatments are best), (d) therapeutic conservatism (less is more), and (e) humanism (care for the person not the disease). Yet these components deal with the approach to a person’s individual health solely. Although important such elements narrowly define a profession’s philosophical stance to cover the areas of reform. The narrowness is that it only approaches the person, their anatomy and homeostatic station without consideration to the multitude of factors that have potential impact upon the human condition. Scientific reductionism has created exceptional medical advances but these can create fragmentation and depersonalization of the patient [17–22]. The question becomes “how does the embracing of vitalism or holism impact regulatory reform or Medicare/Medicaid reform?”

Attempts have been made to adopt the biopsychosocial model to fully comprehend the totality of the human condition. The biopsychosocial model (BPS) suggests that states of health and illness can be understood in terms of their biological, psychological, and social parameters [23]. Philosophically, it is a way of comprehending how the states of suffering, disease, and illness are affected from the societal to the molecular [24]. Lindau et al. [25] noted three aspects of the BPS relevant to this discussion. First, a philosophy must address health along with illness. Second, there is an aim to not work through a singular cause of disease and ill health but rather work and decipher through the myriad of biophysical, psycho-cognitive and social processes. Finally, a philosophy must take into account the plethora of individual social networks in order to maximize health for the individual, their family and society [25]. In other words, it goes beyond the person and their health and delves into the psychological and social and hints at the political and economic. The BPS model is relevant for a patient-centered approach yet a philosophy of chiropractic medicine has yet to incorporate these three concepts using principles.

In the effort to formulate principles there is a fundamental need to deal with the socio-political aspect of the human condition and an understanding of the body politic. Kumar and Preetha [26] suggested that many factors influence health and social wellbeing outside the health system. Such factors include socioeconomic conditions, patterns of consumption, demographic patterns, education, family patterns, the fabric of social and culture of societies as well as the sociopolitical and economic challenges [26]. To date, there has been a paucity of commentary introducing principles addressing the sociological, economic and political realms with just a few papers written addressing national policy issues [27, 28]. Yet with policy, there is always politics. Politics can be defined as the “societal mechanism that strives to manage conflicts that are challenging to resolve in which there are disagreements among social groups” [29]. Two types of politics exist: formal and informal. Formal politics deals with the politics of formally identified public institutions and local, state and federal/national governments [30, 31]. An example of this is through the legislative process. Informal politics is defined as the formation of alliances outside of the framework of the three types of government (local, state and federal/national) that exert influence upon the protection and advancement of ideas and ideals [30, 31]. A prime example of this is a protest march for an ideal in which public opinion is hoped to be shaped. The definition of formal and informal politics is applicable to the chiropractic profession and its continued internecine struggle to define itself. Despite this difference, it should be remembered that health is multi-faceted and is involved in both definitions of politics. It is noted that health is dependent upon a highly sophisticated network of many factors ranging from the physical, biological, environmental, economic, social, cultural, spiritual as well as moral [25]. Based on this the need to begin the discussion utilizing a social, political and economic philosophy becomes even more apparent.

### Questions

Many questions arise that are pertinent to the discussion. How does a profession develop principles of practice and policy to address significant areas of reform and concern? What framework can be used to begin the process? How can the profession develop a philosophy that will guide it through the political landscape? What principles can the profession avail itself upon to guide it through socio-cultural issues and establish health, social, political and economic policy? In fact, it would be quite difficult to solve the numerous areas of needed reform with a philosophy promoting integrated components of vitalism, holism, naturalism, therapeutic conservatism, and humanism. It can be argued that the tripartite rationale of evidence-based (best available evidence, patient preferences and individual practitioner expertise), outcome driven, and patient-centered, exist as the modus operandi of chiropractic medicine’s clinical philosophy. Yet, this has had little impact upon political, economic and social policy beyond the practice of health care. This difficulty is appreciated because it does not delve into the real issues affecting a nation from the sociological, economic and political standpoint and for effective delivery of chiropractic medicine. A viable philosophy based on principles can guide the current professional advancement in a proactive way.

Vidal [32] noted six fundamental questions that are applicable to a PCM. These questions are: (a) what is…? (b) where does it all come from? (c) where are we going? (d) what is good and what is evil? (e) how should we act? and (f) what is true and what is false? Such models of ontology (model of reality as a whole), explanation (model of the past), prediction (model of the future), axiology (theory of values), praxeology (theory of actions) and epistemology (theory of knowledge) are of vital import to the development of a PCM [32]. To date, Vidal’s [32] questions have yet to be adequately answered such as “what is chiropractic medicine?” and “where is chiropractic medicine going?”. While these questions need to be answered, a framework of principles is required by which to assist in formulating an adequate response.

With policy, along with development and impact of such, being even more on the consciousness of the common citizen, the need to formulate sound principles is needed to develop good policy. The addition of the BPS model adds much to the philosophical approach to the doctor-patient encounter. But even this addition does little for establishing core principles for a PCM that will guide the profession in communicating to potential patients and policy makers on where it stands on key social, economic and political issues. Nelson et al. [1] detailed a set of criteria for chiropractic as a profession. These criteria are comprised of being consistent with accepted modes of scientific reasoning with the need to accommodate future changes of scientific reasoning and knowledge [1]. Arguably, any criteria put forward will need to be credible and communicable to external constituencies and have a substantial presence within the healthcare marketplace [1]. In order to piece together a movement to propose such a model requires coordination between people and the formation of organizations. Yet, organizations, by their very nature, are political systems whether they claim such or not [33]. A philosophy which can guide an economic and social policy, embrace science, accommodate future scientific thinking, be skeptical of findings, be credible and communicable to potential health care consumers needs to be considered that can be accepted by not only external constituencies but to internal constitutes as well.

### A shift in thinking and purpose

In attempting to propose a framework for a PCM, there will be a need for a paradigm shift of thinking. The shift in thinking for the reader will come in the form of not thinking in the context of clinical issues and matters of the profession but more about policy issues that can impact a nation via social, political and economic policy. What may be viewed as tangential issues with each principle presented requires the shift in “how to think and apply chiropractic medicine” to the larger issues affecting a nation.

In order to address the concerns presented by Murphy [13, 14], Walker [15] and Ausman [16] the primary purpose of this paper is to expand upon the work of Russell Kirk (b.1918, d. 1994), an American political theorist, as a basis for introducing philosophical principles. A further purpose is for the development of a PCM and begin the process, through debate and discourse, of developing principles for not only the approach to the human condition but for the progression of a profession that will impact social, political and economic policy. To date, no other profession has begun such a process of utilizing the principles presented in this paper.

Kirk’s Ten Conservative Principles provides the necessary framework for several reasons. Kirk [34] sought to set the individual person free from their own bias and become more thoughtful about their self-expressed views. Furthermore, these principles argue for what is “a state of mind, a type of character and a way to examine the civil social order” [34]. Such principles provide a guide to policy formulation far better than the esoteric meanderings of coffee shop philosophers [34]. Finally, it accommodates for the wide ranging diversity of viewpoints on many complex subjects [34] with there being no dogmatic premises such as a Thirty-Three Principles as espoused by Stephenson [35]. In a polarized political world some may be concerned about the term “conservative.” Yet, this is merely an adjective with no real implication [34]. Most will agree, no matter what the political spectrum one hails from, individual chiropractors should (a) place principles and ideas above one’s own personal desire, (b) recognize and utilize the benefits of hard work and competition, (c) promote self-help rather than dependence on government and others, (d) emphasize self-reliance, (e) be able to reap the fruits of one’s labor, (f) taking practical action to improve one’s situation and (g) emphasizing humility and open-mindedness [36].

## Discussion

### The core principles of Kirk

Russell Kirk (b.1918, d. 1994) was an American political theorist. Kirk [34, 37, 38] is known to have written and spoken on issues such as modern culture, political thought, educational theory and social themes. The Ten Conservative Principles comes from a speech delivered by Kirk for the Heritage Foundation in 1986 [37]. Kirk [37, 38] intended his ten principles to be a “body of sentiments, rather than a system of ideological dogmata”. These ten principles that can form the necessary framework for philosophy of chiropractic medicine include: (a) moral order, (b) custom, convention and continuity, (c) prescription, (d) prudence, (e) variety, (f) imperfectability, (g) freedom and property linkage, (h) voluntary community and involuntary collectivism, (i) prudent restraints upon power and human passions, and (j) permanence and change [37, 38]. Each of these principles will be elaborated upon as it applies to chiropractic medicine. Table 1 provides an overview of the principles and a brief definition of each principle. Table 2 provides the core principles of Kirk and explanations applicable for a PCM.

**Table 1:** Principles and brief definitions as established by Kirk [37, 38]

Principle	Definition
Moral Order	Human nature is a constant, and moral truths are permanent.
Custom, Convention, and Continuity	Custom: enables people to live together peaceably.
	Convention: contrive to avoid perpetual disputes about rights and duties
	Continuity: the means of linking generation to generation.
Prescription	Things established by immemorial usage, so that the mind of humans do not run to the contrary.
Prudence	Public measures ought to be judged by its probable long-range consequences, not merely by temporary advantage or popularity.
Variety	Affection for the proliferating intricacy of long-established social institutions and modes of life.
Imperfectability	Human nature suffers irremediably from certain grave faults.
Freedom and Property Linkage	Freedom and property are closely linked.
Voluntary community and Involuntary Collectivism	A successful spirit of community is made locally and voluntarily.
	A distant political direction that is centralized and uninterested can become hostile.
Prudent restraints upon power and human passions	A just government maintains a healthy tension between the claims of authority and the claims of liberty.
Permanence and Change	The Permanence of a society is formed by those enduring interests and convictions that give stability and continuity.
	Progression is that spirit and that body of talents which urges one toward prudent reform and improvement.

**Table 2:** Principles of Kirk [37, 38] as applicable to a philosophy of chiropractic medicine (PCM)

Principle	Definition
Moral Order	Moral truths are consistent, guided by sound ethics, for patient-care and professional behavior. These moral truths are supported for people to live in a peaceful society.
Custom, Convention, and Continuity	A recognition and appreciation of long-standing practices, traditions, and respect for institutions of society are vital for the overall well-being of a nation.
	Contriving to avoid perpetual disputes about the rights and duties of the patient and the rights and duties of the clinician.
	Current state laws regarding matters of chiropractic interest and for society, while subject to the profession and enacted via laws by the citizenry enables people to live together peaceably through the upholding of law; there exist core aspects of the profession that remain consistent i.e. spinal manipulation and the profession as chiropractic medicine with appreciation for the social contract.
Prescription	There are those in the history of science and medicine that inspire and encourage investigation. The basic tenets of science guide the chiropractic medicine practitioner in order to keep from contrary actions such as pseudoscience. There is respect and regard for a nation’s founders and those who propelled it to greatness and for a nation’s unique culture and history.
Prudence	Public health measures and other measures introduced for the good of society and individuals are judged by their probable long-range consequences, not because they are popular or provide a temporary political advantage. Chiropractic medicine seeks to empower individuals and promote basic freedoms.
Variety	Affection for the established scientific processes that exist; variety in the establishment of innovative methods to deal with neuro-musculoskeletal conditions is encouraged through the scientific method. The chiropractic clinician makes use of additional diagnostic, therapeutic, pharmacologic and rehabilitation modalities to serve a greater population of patients. Chiropractic medicine realizes that society will have all levels of economic classes and that such classifications signifies a healthy society.
Imperfectability	Human nature suffers under its own weight from the psychological to the genetic. Because of this imperfectability, human biological systems break down and need repair thus negating the philosophy of self-healing without choice. Individual responsibility is the key to effective patient care and not dependent upon the doctor to make a patient into a “new healthy”. Acknowledgement that utopia or utopian measures can never come to fruition.
Freedom and Property Linkage	Chiropractic medicine advocates for personal freedom and individual rights of self-determination of the patient and society as a whole. Chiropractic medicine promotes the ownership of personal property, asset and wealth accumulation and responsibility for that property. It also advocates for the constitutionally-guaranteed rights provided by their nation and advocates for property ownership and the individual as consumer. Chiropractic medicine promotes market-based solutions to complex economic issues and incentives for growth of the profession, society and the patient.
Voluntary Community and Involuntary Collectivism	Chiropractic medicine practitioners advocate for individual human rights, achievement, individual responsibility and the power of individual human potential. Chiropractic medicine advocates for the local community to voluntarily empower itself and opposes collectivism that forces individuals and/or groups to the will of an uninterested and unrepresentative entity.
Prudent restraints upon power and human passions	Chiropractic medicine physicians advocate for prudent restraints on the practice of chiropractic medicine. Prudent restraints on what is advertised, practiced, and advocated so as human passions do not impede upon progress. Chiropractic medicine advocates for limited government that does not impede upon personal liberty and promotes the rule of law as no one person is above the law
Permanence and Change	Chiropractic medicine embraces the permanence of those things inherent to the operation of healthcare to ensure stability and continuity; for chiropractic medicine to progress it must acknowledge that change is necessary and healthy.

1. Principle of Moral Order
  The principle of moral order posits that a society in which men and women are governed is by the belief in an enduring moral order. There exists a strong sense of right and wrong, truth and falsehood [38]. These are guided by personal convictions about justice and honor. With this, there is the belief in a foundational moral order (right and wrong, justice and honor) guided by personal convictions that a good society will evolve and continue [37, 38]. Kirk argued that human nature and behavior is a constant and that moral truths are permanent [37, 38]. It is advocated that there exists objective truth in the universe and it can be known [38]. Kirk [37, 38] suggested that in the desire for a moral order that there would be harmony i.e. a harmony by consistency of thought. This harmony does not preclude that all things that one confronts be in congruency and cannot be meant to signify “harmony of nature” since it would behoove the philosopher of believing that there is harmony or order in the universe [37, 38].
  Many professions pronounce what a profession’s ethics are yet seemingly fail to elucidate what makes for unethical behavior. Brown [39] recognized that there must be a strong and robust effort to place condemnation of unethical practices that fall below acceptable standards. There currently exists a public image that is unfavorable toward the chiropractic profession as it pertains to ethics and honesty [40]. The Principle of Moral Order is entwined in ethics. With ethics, it is interpreted that this principle serves as an anchoring principle for a PCM.
  Kirk [37, 38] further defined two types of order: (a) the inner order of the soul and (b) outer order of the commonwealth (a state where the supreme power is vested of the people, for the people and by the people). A PCM can cover both of these orders. The inner order of the soul is distinctively the ethics that a practitioner must hold dear. The principle of moral order of the inner soul does not proffer that one follows a religious sect or particular theological denomination of thought. Thus, there is freedom to pursue the religion, theology and spirituality that one chooses to follow reigns supreme here as well as the acknowledgement of a higher power or God. For example, a person can claim to be a scientist yet hold the view that God exists.
  The outer order of the commonwealth is uniquely suited for the chiropractic profession. A PCM should hold a concern not only for how one acts/behaves with the professional responsibility but with the concern for the future health of the profession. It should be of further note that the outer order is not simply for the future of the profession solely. A pride and appreciation for one’s own country, direction, belief in their nation’s exceptionality, constitutional form of government, sovereignty, freedoms, accomplishments, language and preservation of its unique culture, heritage and history are manifest in this principle.
  The basic definition of this principle that is applicable for a PCM would be stated as: *Moral truths are consistent*, *guided by sound ethics*, *for patient*-*care and professional behavior. These moral truths are supported for people to live in a peaceful society*. In the quest to reform the profession, this may be the one of the more important principle to address. In fact, this encourages the answering of Vidal’s [32] question of “what is good and what is evil?” Furthermore, this principle addresses Walker’s [15] concern of individual personal leadership to effect change and Ausman’s [16] concern about the loss of ethics and morality.
2. Principle of Custom, Convention, and Continuity
  Kirk [37, 38] determined three aspects of human life that were essential: custom, convention and continuity. These three aspects are necessary ingredients for the sustaining of societies. Each of these has pertinence and applicability to the development of a PCM.
  Custom enables people to live together peaceably [37, 38]. Traditional values among groups, social and political customs that have been passed down from one generation to the next generation enable people to live together peaceably [37, 38]. Within the context of a PCM, customs are defined as a “unified etiquette” such as privacy and personal space. In clinical medicine, the concept of the doctor-patient relationship is one of “trust” and in a non-authoritarian attitude. The Hippocratic Oath, doctor-patient confidentiality, and the use of the caduceus are all examples of custom. Thus, customs in medicine have been practiced for a considerable time and remain stable fixtures. Among various social groups, the attitude of patients toward a clinician is as much a cultural component as is family and church to some social groups [41–46]. These customs are essential to how people live peaceably.
  With convention, there is a constant need to deliberately create rather than to assume that things will arise naturally or by spontaneous means [37]. By deliberate creation, thus come the end-product of the avoidance of constant and never-ending disputes about individual rights, duties and responsibilities [37]. Law, at its foundation, is a body of conventions [38]. A PCM follows convention by following law as well as influencing law-making via voting and lobbying efforts. The history of chiropractic and its effort to gain legal recognition is an example of convention i.e. the deliberate creation of a profession through legal means and with attempting to end questions about duties and responsibilities.
  Continuity refers to the means of linking generation to generation [37, 38]. This is ultimately the telling of one’s history that is perpetuated similar to that of a family or shared cultural history. A PCM embraces this continuity in that through historical coursework the history of the profession is shared thus linking each new generation of chiropractors who did not experience that which others were challenged. An example of continuity can be appreciated in the fidelity to the social contract. Menand [47] wrote: “Professions are largely self-regulating; they set the standards for entrance and performance in their specialized areas and they do so by the light of what is good for the profession rather than what market conditions or external forces, such as legislators or citizen groups, demand.” A certain degree of autonomous control over a profession by the individuals in a profession has been granted by society. However, this social contract demands that each profession and professional place the wellbeing of society and the patient, client or parishioner ahead of their special interests.
  The basic definition of this principle that is applicable for a PCM would be stated with regards to custom: *a recognition and appreciation of long*-*standing practices*, *traditions*, *and respect for institutions of society are vital for the overall well*-*being of a nation*. As it pertains to convention a PCM would define this as the *contriving to avoid perpetual disputes about the rights and duties of the patient and the rights and duties of the clinician*. Furthermore, this principle suggests that *current state laws regarding matters of chiropractic interest and for society*, *while subject to the profession and enacted* via *laws by the citizenry enables people to live together peaceably through the upholding of law*. With continuity the definition would be that *there exist core aspects of the profession that remain consistent* i.e. *spinal manipulation and the profession as chiropractic medicine with appreciation for the social contract*. These three aspects of the Principle of Custom, Convention, and Continuity are applicable to Walker’s [15] suggestion for establishment of a progressive identity.
3. Principle of Prescription
  Kirk [37, 38] defined the Principle of Prescription as those things established by immemorial usage, so that the minds of humans do not run to the contrary. The spirit of this principle is that one’s greatness as a profession and nation comes from the hard work and inspiration of those who have come before [37, 38]. Kirk [37] believed this concept as “resting on the shoulders of giants.” In other words, there is honor of and for the pioneers, innovators and patriots. It is also a call to remind one to hold regard for the established biomedical and sociological, economic and political principles set forth by the pioneers, innovators and patriots in order not to fall into a contrarian point of view. The history of biomedicine is filled with those whose contributions are still utilized today and serve as an influence to chiropractic medicine. Scientists such as Newton (mathematics, law of gravity, and the Laws of Motion), Roentgen (x-ray), Pasteur (pasteurization), Vesalius (anatomy), Lavoisier (metabolism), Hippocrates (father of modern medicine), Popper and Kuhn (philosophy of science), Drew (blood banks), Sackett (evidence-based medicine) and many more are regarded immemorial for their contributions. Their contributions can be directly attributed to our modern understanding and development of chiropractic medicine. This principle can be expressed for a PCM as: *there are those in the history of science and medicine that inspire and encourage investigation. The basic tenets of science guide the chiropractic medicine practitioner in order to keep from contrary actions such as pseudoscience. There is respect and regard for a nation*’*s founders and those who propelled it to greatness and for a nation*’*s unique culture and history*. This principle has direct application to Walker’s [15] admonishment to embrace an evidence-based practice and a pro-public health stance. It also supports Murphy et al. [13, 14] suggestion for research and education.
4. Principle of Prudence
  The Principle of Prudence derives its origin from Plato’s *Republic* of the four cardinal virtues (prudence, justice, temperance, and courage) [37, 38]. Kirk [37, 38] defined this principle as “any public measure ought to be judged by its probable long-run consequences, not merely by temporary advantage or popularity”. Society, because of its complex nature, requires solutions that are not simple if such solutions are to have any efficacy [37, 38]. This principle states that one acts upon any measure after there is sufficient reflection as well as a careful weighing of the potential consequences of any action [39, 48]. Under the Principle of Prudence items of interest need to be judged by the item’s probable long-term consequences and not for political convenience. Simply, this principle suggests that one does what works, not what sounds good nor what is politically expedient and/or popular. One has to see the world as it exists, not see the world how one wishes it to be.
  Efforts at public health, self-empowerment, integration, clinician and professional autonomy are affected by this principle. The Principle of Prudence has direct connection to many aspects of patient care for a PCM most notably that of public health. Murphy et al. [49] indicated that public health is ultimately about individual self-empowerment. It is about the informing of people on how to take care of themselves and emphasizes two aspects: prevention and health maintenance [49]. Yet there are freedoms by which to inform people and freedoms by which to follow advice. A PCM advocates that clinicians be contributors to sensible health information and not propagators of misinformed beliefs or spurious speculation. As educators of healthcare there is a responsibility that must be taken seriously, for the implications of errors of such misinformation can be significant.
  For self-empowerment, individual freedom must be appreciated and realized with personal responsibility prevailing. In other words, a PCM would advocate that personal responsibility and individual freedom go hand-in-hand. With self-empowerment, dependency on any system should not occur for the long-term unless the person is rendered powerless based on disability, handicap or impairment. With dependency a “culture of dependency” can spread through not only a family, but for local business, community, and even society [50]. This type of culture will not only create an emotional hardship but a financial burden for the society-at-large [50]. Short-term measures as a safety net should be advocated in order for people to fulfill their individual right of self-empowerment toward self-determination.
  For a PCM, this principle is expressed as *public health measures and other measures introduced for the good of society and individuals are judged by their probable long*-*range consequences*, *not because they are popular or provide a temporary political advantage. Chiropractic medicine seeks to empower individuals and promote basic freedoms*. The principle is directly applicable to Walker’s [15] encouragement for a pro-public health stance. It supports Murphy’s et al. [13, 14] need for education, regulatory and research reform. Furthermore, it relates to Ausman’s [16] concern about an entitlement mentality and loss of freedoms.
5. Principle of Variety
  Kirk [37, 38] defined this as “the affection for the intricacy of the many modes of life and affection for a cherishing of long-established social institutions.” With the respect for affection of long-established institutions (family, religion, education, media, law, politics/government, economics, science, military, and medicine), it is intended that this principle avoid uniformity that narrowed the human condition [37, 38]. It is also interpreted that there can only be two types of equality: equality in a court of law and at the Last Judgement [37, 38]. There will always be inequality in many forms no matter what because if all modes are considered equal then all modes of life become uniform thus leading to stagnation [37, 38]. It is interpreted that this principle is to mean that there will always be gaps among people’s earnings and is believed part of a healthy society. There will always be an upper class, a middle class and a lower class in the socio-economic scheme of things. There is a need for a healthy diversity in any society in that orders, classes, differences in material wealth, and differences of opinion will always exist [37, 38]. Since capitalism is designed to promote productivity, it can be expected to have inequalities of income and wealth [51]. Cole [52] noted that “given the observed diversity among human beings, in tastes and preferences, in talents and capacities, it should come as no surprise that the logic of income distribution under capitalism results in significant inequality of money incomes”.
  Chiropractic medicine, while argued by some to be an advocate for social justice, [27, 28] needs to have the realization that a healthy society has class differences and that there will exist injustices no matter what economic, political or sociological system is in play. This principle is indicative that there is an appreciation to advocate for citizens to be able to move from current sociological or economic stations toward higher levels of prosperity and quality of life.
  Evidence-based medicine (EBM) provides an example of the Principle of Variety. EBM was devised based on three foundational pillars: best available evidence to make a clinical decision, patient preferences for care and individual clinician expertise. Yet it was argued by chiropractors that it would be a death-knell to chiropractic. The argument was that if clinical expertise is absent, then practice risks would be tyrannized by the best available evidence solely. It was believed that evidence-based practice denigrated clinical judgment and experience. Further, evidence-based practice was argued that it would lead to a “cookbook” practice style and all patients would be treated the same no matter the peculiarities of their unique situation [53]. At first glance, it would appear that such would stagnate variety. However, innovation and variety were essentially heightened as it is a model in which much interpretation and skill were needed to utilize the model.
  Another example of the Principle of Variety is demonstrated. Brown [30] argued that significant variation in chiropractic education as well as practice patterns and approaches can present an obstacle to widespread acceptance. The Principle of Variety has limits. The Principle of Variety does not mean a laissez-faire approach of “anything goes” or that “all techniques work” to clinical practice or that a large variation in practice styles is an asset. Chiropractic medicine realizes that the variant in chiropractic clinical medicine is in the provider, not the paradigm of treatment. In other words, while two practitioners may have the same clinical training, provide the same treatment focus for a certain condition, the variety therefore becomes the “art” of the delivery of services. Thus, the humanistic approach, i.e. bedside manner and skill, that varies from practitioner to practitioner, becomes the free market reality.
  Although the Principle of Variety as defined by Kirk appears to be discussing society from a class perspective, it can also be interpreted that variety is a healthy form for the chiropractic medicine profession. By combining many forms of treatment modalities what is best for the patient with the best available evidence, along with clinical reasoning (and to a certain extent, creativity and improvisation), variety can take place. For chiropractic medicine, this principle can be expressed as: *Affection for the established scientific processes that exist*; *variety in the establishment of innovative methods to deal with neuro*-*musculoskeletal conditions is encouraged through the scientific method. The chiropractic clinician makes use of additional diagnostic*, *therapeutic*, *pharmacologic and rehabilitation modalities to serve a greater population of patients. Chiropractic medicine realizes that society will have all levels of economic classes and that such classifications signifies a healthy society*. The Principle of Variety is directly applicable to Ausman’s [16] concern about the socialization of America and an entitlement mentality and the concern about the deterioration of the economy. This principle has significance for Murphy et al’s [13, 14] advice for regulatory reform and marketing reform.
6. Principle of Imperfectability
  The human body in all its beauty and functionality is prone to failure. Human nature suffers irremediably from many grave faults thus no perfection can be appreciated [37, 38]. This principle is applicable to chiropractic medicine based on the association with the spectrum of the human condition. The seven dimensions of wellness (social, emotional, spiritual, environmental, occupational, intellectual, physical) are examples of the human condition of being imperfect. For example, if the intellectual dimension was not prone to error, no one would ever fail an examination. By this, the Principle of Imperfectability strongly counters any type of metaphysical aspect self-healing of the human biological system or any thinking that believes that humans are capable of perfection. It also strongly counters the notion of placing individuals into an ideal health state or creating a society based on utopianism. Individual responsibility is at the core of this principle.
  As mentioned previously, what the Principle of Imperfectability strives for is the guarding against any utopian ideal. Kirk [37, 38] noted that “man being imperfect, no perfect social order ever can be created”. Due to human restlessness, people would grow rebellious under any type of domination via a utopian ideal [37, 38]. Throughout history there have been those who have aspired to create utopian societies but have done no more than create earthly wastelands [37, 38]. Even the founding fathers of the United States intuitively knew of such when devising the concept of “the more perfect union” in that no perfection can be obtained.
  Health is, by and large, a part of the political life of the world. Considering that one-sixth of the American economy is dedicated to matters of health, it would be incorrect to state that health is apolitical. Health is political based on the knowledge that power is exerted over it as part of the wider social and economic system [54]. Bambra et al. [55] brought forth five types of health: (a) health as an ideal state, (b) health as a personal strength or ability, (c) health as physical and mental fitness to do socialized tasks, (d) health as a commodity, and (e) health as the foundation for achievement of potentials. A PCM would need to devise a definition of health that follows along the lines of the principles discussed in this paper. Each of Bambra’s [55] types may be considered worthy yet each has strengths and pitfalls. For example, if health is considered an ideal state, the urge to make everyone fit an ideal state can become tyrannical. An example could be argued that the Body Mass Index chart has caused people to feel guilty about themselves. Thus, the aspiration to an ideal health state can be worthy but knowledge of the Principle of Imperfectability precludes it from becoming actually idealized. Defining health needs to ensure that utopian ideals do not come forth yet allows individual self-empowerment and to extenuate the power of individual human potential should be a mainstay of chiropractic medicine.
  This principle can be expressed for a PCM as *human nature suffers under its own weight from the psychological to the genetic. Because of this imperfectability*, *human biological systems break down and need repair thus negating the philosophy of self*-*healing without choice. Individual responsibility is the key to effective patient care and not dependent upon the doctor to make a patient into a* “*new healthy*”. *Acknowledgement that utopia or utopian measures can never come to fruition*. This principle has congruence with the Principle of Moral Order. Furthermore, this principle has policy significance for establishing not only a philosophical explanation similar to that Coulter’s [2] models but can be linked to Walker’s [15] suggestion that there be a marginalization of elements of nonsense within the chiropractic profession.
7. Principle of Freedom and Property Linkage
  Kirk’s [37, 38] principles do not specifically define an economic philosophy. Yet there is intricate link with the Principle of Freedom and Property Linkage as the basis for an economic philosophy. A PCM will need to understand the intricate nature of an economy of chiropractic medicine. These issues are raised for the discussion to ensue about the direction of a philosophy of policy towards economics that impact the chiropractic medicine profession, practitioner and ultimately the patient and society. The basis of Kirk’s [37, 38] principles is that freedom and the ability to own personal property are intricately linked together. The inability to own property would create a national government (state) to be unstoppable [38]. People need the ability of ownership to secure individual rights, become better and responsible citizens and limit the power of government [38]. The ability of individual ownership of property, a person is able to rise from the natural condition of poverty to what is known as the “security of enduring accomplishment” [37]. Kirk [37] believed that economic levelling is not economic progress. Kirk noted [37]: “Separate property from private possession, and Leviathan becomes master of all. Upon the foundation of private property, great civilizations are built. The more widespread is the possession of private property, the more stable and productive is a commonwealth.” For purposes of this section, there is need for clarification of classic economic systems. Ausman [16] presented two types of economic systems: a centrally-controlled economy and a free market economy. With a centrally controlled economy as proposed by John Maynard Keynes (Keynesian economics), that during times in which the economy slowed, the government should print money so that the public would be able to buy goods to overcome the crisis [16]. With a free market economy, as proposed by Friedrich Hayek, support for the free market economy reigns and not government intervention into markets or the publics’ choices [16]. These two economic systems have pertinence for how a PCM should approach economics. Such topics as government regulation, socialized medicine, the creation of a viable economic model for chiropractors, and entrepreneurialism are encountered. The Principle of Freedom and Property Linkage should be considered by a PCM as the driver of economic policy for chiropractic medicine.
  Chiropractic physicians have been, by history, small business owners. Murphy et al. [50] reported that market share dwindled from 10% of the population to 7.5%. In a ten-year period (1987 to 1997) amongst patients with back pain, the proportion of patients seeing chiropractors dropped significantly [50]. These statistics are of concern yet there is opportunity. Nelson [1] suggested conservatively that at least 75% of spine care patients potentially could stand to benefit from chiropractic care while only 12–17% of spine care patients currently avail themselves to chiropractic spine care. Between 1999 and 2008 the mean inflationary adjusted costs for ambulatory neck and/or back pain in the United States increased by a factor of 95% [56].
  Although these statistics [1, 56] offer potential opportunity for chiropractors there exist a larger picture that is missing from the discussion. The private sector labor force produces the majority of American jobs, goods, services and revenue needed to sustain economic growth [57]. The Economic Policy Institute [58] indicated that there were some 3 million missing workers in the labor force which would make the current unemployment rate in the United States (U.S.) at 6.5%. According to Vollmer [57], in the U.S., 112 million private sector workers support 32 million government workers and contractors, 94 million able-bodied people who can work but chose not to work [59], 70 million who cannot work and the 16 million unemployed. Of the 112 million employed Americans in the private sector, approximately 60% are standard full-time workers and 40% are part-time and independent contract workers [57]. Thus, the U.S. economy cannot be sustained by only 34% of the population that is eroding in terms of size, wages and income potential [57]. As well, there is $2 trillion dollars collected in taxes each year in the U.S. and over $3.5 trillion dollars being spent each year [16]. There has been a decline of “entrepreneurial activity” from 14% in 2014 to 12% in 2015 [60] with the labor participation rate at 62.7% as of August, 2016 [61]. Real household income levels have declined from $57,000 in 2007 to $53,700 in 2014 [60, 62]. With the decrease in household income and less people working, the home ownership rate has declined to a near 48-year low [60].
  These numbers alone indicate that an economic philosophy needs to be included in a PCM. Embracing economic policy that will promote and create jobs, increase individual income, decrease tax burdens, decrease punitive restrictions and regulations on individuals and business, promote deficit reduction, and stimulate national economic growth will go far in forwarding chiropractic medicine and society as chiropractic patients are citizens. With such a principle, the investment in entrepreneurship, a focus on tax policy, the patient as consumer, and other items become mainstays for a PCM. For a PCM, the Principle of Freedom and Property Linkage can be expressed as: *chiropractic medicine advocates for personal freedom and individual rights of self*-*determination of the patient and society as a whole. Chiropractic medicine promotes the ownership of personal property*, *asset and wealth accumulation and responsibility for that property. It also advocates for the constitutionally*-*guaranteed rights provided by their nation and advocates for property ownership and the individual as consumer. Chiropractic medicine promotes market*-*based solutions to complex economic issues and incentives for growth of the profession*, *society and the patient*. The Principle of Freedom and Property Linkage can be directly related to Ausman’s [16] concern about the progressive centralization of the control of medicine by large organizations and the government, concern about the socialization of America and an entitlement mentality, and concern about the deterioration of the economy. This principle also has pertinence to Murphy et al’s [13, 14] call for regulatory reform.
8. Principle of Voluntary Community and Involuntary Collectivism
  The Principle of Voluntary Community and Involuntary Collectivism is related to how people operate within communities. In a genuine community, the decisions most directly affecting the lives of citizens are made locally and voluntarily [37]. From this, the adage that “all politics are local” comes to bear. Included in this ability are not only local political bodies but private associations [37, 38]. Kirk [37] believed this constituted a healthy community. But a distinction needs to be made between voluntary community versus that of involuntary collectivism. Collectivism is the world view which believes that selected elites or “anointed” members of society possess superior knowledge as they should be the ones who will engineer the society based on schemes of central planning [63]. Under the system of collectivism, the average citizen becomes simply “a mere cog in the wheel of the state” [63]. The system of collectivism is involuntary as the citizen is forced to comply to the will of the state without any or adequate representation. The centerpiece of the collectivist egalitarian state (forced division of wealth by the power of the state) is the welfare establishment which serves two purposes: (a) people must be made dependent on government, and (b) the punishing of the individual person of wealth because the state simply sees the individual as the non-compliant enemy [63]. In a collectivist state, the common person’s views, personal plans and desires must be subordinated to that of the grand designers of the state [63]. However, when local functions pass by either default or usurpation to some centralized authority, it is believed that a local community will be in much danger [37, 38]. Those individuals who uphold a voluntary community posture would oppose forms of involuntary collectivism [37]. McLeod [38] noted that “a centralized administration with a corps of unselected managers and civil servants, no matter how well intentioned and well trained, would be unable to confer justice, prosperity and tranquility upon a mass of men and women if deprived of their old responsibilities.” Excellence and high standards and the freedom to achieve high standards provide safeguards to a nation from an overbearing and overzealous collectivism by making it more difficult to standardize people [38]. Furthermore, local voluntary associations and institutions draw humans out and engage people in the community in order to provide a buffer between people and the state [38]. With local voluntary association, the individual is allowed a say. An individualist culture is defined as “a culture in which individual goals take precedence over the group goals” [64]. A collectivist culture, on the other hand, is defined as “a culture in which group goals take precedence over individual goals” [64]. An individualist culture tends to prosper economically whereas collectivist cultures tend to be less prosperous in economic terms [64]. If a PCM claims to promote individual responsibility and self-empowerment, individualist culture needs to acknowledged and encouraged.
  A PCM using the Principle of Voluntary Community and Involuntary Collectivism intricately linked with Principle of Freedom and Property Linkage can economic policy by chiropractic medicine be established. Thus, the principle is expressed as: *chiropractic medicine practitioners advocate for individual human rights*, *achievement*, *individual responsibility and the power of individual human potential. Chiropractic medicine advocates for the local community to voluntarily empower itself and opposes collectivism that forces individuals and*/*or groups to the will of an uninterested and unrepresentative entity*. The use of these principles are supportive of Walker’s [15] efforts to legitimize chiropractic i.e. support for legitimate chiropractic organizations and professional leadership development and Ausman’s [16] concern for current state of the economy, the socialization of America and an entitlement mentality and the loss of individual freedom.
9. Principle of Prudent Restraints Upon Power and Human Passions
  The Principle of Prudent Restraints upon Power and Human Passions is based on the concept that “a just government maintains a healthy tension between the claims of authority and the claims of liberty” [37]. As Lord Acton believed: “power corrupts, absolute power corrupts absolutely.” This principle describes power as “the ability to do as a person likes regardless of the will of one’s peers” [37]. But Lord Acton further indicated: “liberty is not the power of doing what we like, but the right of being able to do what we ought.” It is also suggestive that an all-powerful state consisting of individuals/small groups who are able to dominate the will of other people without any checks or balances would be nothing short of despotism [37]. Kirk [37] intended this principle to arrange government and society in a special way to avoid anarchy and tyranny with the use of constitutional checks and balances. Along with this is the adequate enforcement of the laws with an intricate system of restraints upon the will and appetite of individuals as instruments of law and order [65]. But since governments are made of fallible humans and are not always well-intended, governments also must be limited [66]. In economics, there exists the Principle of Maximization that is suggestive that all individuals are always motivated by greed [67]. The Principle of Prudent Restraints applies the principle of limited government based on the rule of law to every proposal as no one person is above the law. It also applies to the private sector in that law and ethics are the brakes by which to limit human passions.
  Nelson et al. [1] and Homola [68] have pointed out that there exist questionable institutionalized practices prevailing in the chiropractic profession. One of the main institutionalized practices was one relating to the practice management industry [1]. While one can believe in unfettered capitalism, questionable practices can harm the profession if self-limitation is not heeded i.e. following the responsibilities of the social contract. Failure to reign in these types of practices and control the powerful passions of fallible humans, unwanted government-mandated limitation may come forth (involuntary collectivism). Thus, a PCM must confront prudent restraints on power within the profession that can become ethical issues.
  However, with chiropractors being more aligned with the private sector, there is the need to limit governmental actions. Government regulation has brought about the recognition of the full and increased costs of doing business [69]. It has been observed that today most rules and regulations that seemingly function as laws are not made by the legislature but rather by uninterested bureaucracies from a central location. For numerous programs run by the federal government it takes taxpayers’ money, runs it through a bureaucracy, and then distributes what is left of it to state and local bureaucracies [70]. From this, it has been estimated that about 35 cents of every dollar spent on some government programs go to the intended beneficiaries [70]. In the attempt to reduce social inequalities in health, there will be greater regulation of products and services that have an impact over health [71]. For example, a government program to eliminate carbon pollution may spill over to include anyone who has a backyard barbeque grill [72]. Although a caricature the point being that what may be a noble public health goal may become a noble violation on privacy rights. Operating by regulatory fiat instead of from law-making bodies imposes not only financial cost but an imprudent use of governmental power.
  While over-regulation may be a problem, lack of regulation can turn into horror. History is replete with incidents and accidents, due to human error and a lack of rules and regulations, that failed to take into consideration personal safety [73, 74]. In other words, in some cases, profits were the rule at the expense of individual welfare and employees. The type of over-regulation discussed here are those that creates increased cost to the employer at the expense of the common citizen but not at the expense of safety. A PCM should and must be for regulation, yet argue to maintain a posture against those regulations that create a stifling effect on productivity, profit and personal liberty.
  In a PCM, there exist a healthy tension between the authority of government and that of individual human rights. A PCM in its advocating of and for public health and other measures needs to follow the Principle of Prudent Restraints upon Power and Human Passions. Thus, there is a linking of the Principle of Prudence with the Principle of Voluntary Community and Involuntary Collectivism. The Principle on the Prudent Restraints upon Power and Human Passions is expressed for a PCM as *chiropractic medicine physicians advocate for prudent restraints on the practice of chiropractic medicine. Prudent restraints on what is advertised*, *practiced*, *and advocated so as human passions do not impede upon progress. Chiropractic medicine advocates for limited government that does not impede upon personal liberty and promotes the rule of law as no one person is above the law*. There is little doubt that this principle has applicability for Walker’s [15] marginalization of elements of nonsense in the chiropractic profession. It also has direct application to Murphy et al. [13, 14] admonishment for practice management reform and regulatory reform.
10. Principle of Permanence and Change
  A healthy society, in the view of Kirk [37, 38], was one that was influenced by two important factors: permanence and change. Kirk [37, 38] defined the Principle of Permanence as “a society that is formed by those interests and strong convictions that give the society stability and continuity such as institutions and traditions.” Without these strong convictions, institutions and traditions, a society can be broken up thus slipping into a state of anarchy [37, 38].
  Yet, there are those things that are considered “old” in the profession that can be held onto and admired. There are numerous categories of such that encompass this possession of importance. The history of struggle for licensure, development of chiropractic schools that are private and independent, private practice autonomy, state licensure, national and state trade associations, development of a singular national accreditation entity as well as independent scientific journals. These things are held in the possession of chiropractic solely as part of being autonomous. This effort for autonomy and self-determination as well as continued existence is both admirable and worthy. It also demonstrates that there is a seriousness to the responsibility that the public has entrusted. These things provide to people, as brought forth by Kirk [37, 38], the necessary stability and continuity. The PCM regards this Principle of Permanence as not only necessary for the continued advancement of the profession but it is what creates the exceptionality of the profession. There is also the appreciation for the things that have been accomplished.
  However, it is realized that a healthy society needs change in order to survive. The Principle of Progression (change) in a society is the spirit of humankind that has talents and the need for continuous exploration [37, 38]. These body of talents urge a society forward toward prudent reform and improvement [37, 38]. A society that lacks progression will ultimately stagnate. The Principle of Progression dictates that “nothing in society should be wholly old” as well as “nothing should be wholly new” [37, 38]. With this principle, there is an appreciation in a balance of prudence in maintaining what is old that has value and those things that are new have value. Both of these principles are wholly suitable for a PCM. Due to the calls for reform the Principle of Progression is needed in the chiropractic profession.
  But the Principle of Progression has limitations. This limitation is as Rowling [75] stated: “progress for progress’s sake must be discouraged, for our tried and tested traditions often require no tinkering. A balance, then, between old and new, between permanence and change, between tradition and innovation because some changes will be for the better, while others will come, in the fullness of time, to be recognized as errors of judgement.” A PCM must be able to hold to the Principle of Progression but be cautious as to those things that threaten the Principle of Permanence. Thus, this principle is expressed as *chiropractic medicine embraces the permanence of those things inherent to the operation of healthcare to ensure stability and continuity*; *for chiropractic medicine to progress it must acknowledge that change is necessary and healthy*. The adoption of this principle has the potential for meeting all the areas of reform and concern by Murphy et al [13, 14], Walker [15] and Ausman [16].

## Conclusions

A philosophy of chiropractic medicine (PCM) can stand on principle. It (chiropractic medicine) needs to develop and communicate principles for which can resonate with the general populace that includes not just health alone but the social, political and economic life of the patient, profession and nation. This paper is an attempt to elucidate principles for the chiropractor to function fully in chiropractic medicine that goes beyond the tripartite rationale of patient centered, outcomes driven and evidence based and leading to policy development. It is a call to begin the debate and discussion of revitalization of principles for the chiropractic profession. Future commentary should seek to expand upon each principle and demonstrate applicability specifically to the areas of needed reform.

## Abbreviations

BPS: Bio-psycho-social
EBM: Evidence-based medicine
PCM: Philosophy of chiropractic medicine
U.S.: United States
